# Phenotypic characterization of individuals with *SYNGAP1* pathogenic variants reveals a potential correlation between posterior dominant rhythm and developmental progression

**DOI:** 10.1186/s11689-019-9276-y

**Published:** 2019-08-08

**Authors:** Andres Jimenez-Gomez, Sizhe Niu, Fabiola Andujar-Perez, Elizabeth A. McQuade, Alfred Balasa, David Huss, Rohini Coorg, Michael Quach, Sherry Vinson, Sarah Risen, J. Lloyd Holder

**Affiliations:** 1 Department of Pediatrics, Division of Neurology and Developmental Neuroscience, Baylor College of Medicine, 6701 Fannin St, Suite 1250, Houston, TX 77030 USA; 2 Jan and Dan Duncan Neurological Research Institute, Texas Children’s Hospital, 1250 Morsund Street, Suite 925, Houston, TX 77030 USA

**Keywords:** *SYNGAP1*, Autism, Neurodevelopment, Electroencephalogram, Posterior dominant rhythm

## Abstract

**Background:**

The *SYNGAP1* gene encodes for a small GTPase-regulating protein critical to dendritic spine maturation and synaptic plasticity. Mutations have recently been identified to cause a breadth of neurodevelopmental disorders including autism, intellectual disability, and epilepsy. The purpose of this work is to define the phenotypic spectrum of *SYNGAP1* gene mutations and identify potential biomarkers of clinical severity and developmental progression.

**Methods:**

A retrospective clinical data analysis of individuals with *SYNGAP1* mutations was conducted. Data included genetic diagnosis, clinical history and examinations, neurophysiologic data, neuroimaging, and serial neurodevelopmental/behavioral assessments. All patients were seen longitudinally within a 6-year period; data analysis was completed on June 30, 2018. Records for all individuals diagnosed with deleterious *SYNGAP1* variants (by clinical sequencing or exome sequencing panels) were reviewed.

**Results:**

Fifteen individuals (53% male) with seventeen unique *SYNGAP1* mutations are reported. Mean age at genetic diagnosis was 65.9 months (28–174 months). All individuals had epilepsy, with atypical absence seizures being the most common semiology (60%). EEG abnormalities included intermittent rhythmic delta activity (60%), slow or absent posterior dominant rhythm (87%), and epileptiform activity (93%), with generalized discharges being more common than focal. Neuroimaging revealed nonspecific abnormalities (53%). Neurodevelopmental evaluation revealed impairment in all individuals, with gross motor function being the least affected. Autism spectrum disorder was diagnosed in 73% and aggression in 60% of cases. Analysis of biomarkers revealed a trend toward a moderate positive correlation between visual-perceptual/fine motor/adaptive skills and language development, with posterior dominant rhythm on electroencephalogram (EEG), independent of age. No other neurophysiology-development associations or correlations were identified.

**Conclusions:**

A broad spectrum of neurologic and neurodevelopmental features are found with pathogenic variants of *SYNGAP1*. An abnormal posterior dominant rhythm on EEG correlated with abnormal developmental progression, providing a possible prognostic biomarker.

**Electronic supplementary material:**

The online version of this article (10.1186/s11689-019-9276-y) contains supplementary material, which is available to authorized users.

## Introduction

Neurodevelopmental disorders caused by mutations in single genes such as and fragile X syndrome, Phelan-McDermid syndrome, and Rett syndrome offer unique insight into the pathogenesis of these disorders. One common mechanism among many neurodevelopmental disorders which has been suggested is excitatory/inhibitory imbalance leading to developmental and behavioral phenotypes. *Shank3* knockout mice, for example, modeling Phelan-McDermid syndrome demonstrated reduced spine density as well as decreased miniature excitatory postsynaptic current frequency [1]. In a separate example, deleting *Mecp2* in GABAergic neurons in mice revealed phenotypic similarities to whole-body deletion in mice modeling Rett syndrome suggesting a central role for inhibitory neuronal dysfunction [2]. Finally, *Fmr1* knockout mice demonstrated reduced Gad67 levels in neuronal subsets, indicating reduced GABA production might underlie some of the phenotypes associated with fragile X syndrome [3]. Overall, these data indicate that single-gene mutations can lead to neurodevelopmental disorders through alteration in neuronal excitability.

One recently described single-gene disorder that results in excitatory/inhibitory imbalance is due to de novo loss-of-function mutations in *Synaptic Ras GTPase-activating protein 1* gene (*SYNGAP1*, [4]). The SynGAP protein has a critical role in dendritic spine maturation and synaptic plasticity as described in mouse models of *SYNGAP1* deficiency [5–7]. Localized to dendritic spines in neocortical pyramidal neurons, SynGAP has been found to have a role in NMDAR (*N*-methyl-d-aspartate receptor) activity and AMPAR (α-amino-3-hydroxy-5-methyl-4-isoxazolepropionic acid receptor) trafficking [7, 8]. When NMDARs are activated by glutamate, Ca^2+^ ions enter the postsynaptic space, activating calmodulin-dependent protein kinase II (CaMKII) via phosphorylation and is maintained active via autophosphorylation. CaMKII then activates SynGAP, leading to endocytosis of AMPARs [9]. Heterozygous loss-of-function variants in *SYNGAP1* result in reduced inhibition of the Ras pathway causing AMPAR exocytosis to the postsynaptic membrane [10]. Therefore, AMPAR exocytosis is increased which causes an excitatory/inhibitory imbalance, potentially leading to abnormalities during development.

Heterozygous deficiency of *Syngap1* was shown to increase synaptic neurotransmission in mice and in cultured neurons treated with siRNAs [11]. Conversely, overexpression of *SYNGAP1* in cultured neurons demonstrated a significant reduction in AMPAR-mediated miniature excitatory postsynaptic currents (mEPSCs), AMPAR surface expression, and AMPAR membrane insertion [11]. SynGAP also has multiple isoforms, which exert opposing effects on synaptic function. The α1 isoform has been shown to decrease mEPSC amplitude and frequency, while the α2 isoform increases mEPSC amplitude and frequency [12].

Loss-of-function variants in *SYNGAP1* have been identified in individuals from cohorts with intellectual disability (syndromic and non-syndromic), autism, and epileptic encephalopathy [4, 13–15]. This suggests a tremendous breadth of clinical presentations for individuals with pathological mutations in *SYNGAP1*. Previous studies have reported individuals with *SYNGAP1* deleterious variants and associated phenotypes. Reported phenotypic traits have included nearly universal epilepsy and intellectual disability/developmental delay, as well as variable presence of autism spectrum disorder and physical dysmorphisms [16, 17]. However, in-depth and longitudinal clinical characterization of a cohort of individuals ascertained for pathologic *SYNGAP1* variants has not been undertaken. In this study, we present a cohort of patients with novel *SYNGAP1* variants as well as patients with previously reported variants that expands our knowledge of pathogenic *SYNGAP1* variants. We sought to comprehensively characterize the longitudinal clinical phenotypes in this population to determine the evolving spectrum of neurologic and neurodevelopmental abnormalities. Finally, in analyzing these data, we sought to identify potential elements within the serial neurologic and neurodevelopmental evaluations that could serve as biomarkers for disease identification, progression, and prognosis.

## Methods

This study presents a retrospective review of clinical data from patients with pathogenic *SYNGAP1* variants identified by clinical next-generation sequencing. All participating subjects were identified within a dedicated Primary Synaptopathy clinic at Texas Children’s Hospital in Houston, Texas. Patients were evaluated on one or several occasions over a 6-year period. Any patient with a deleterious *SYNGAP1* variant was eligible to participate. Patients were excluded if additional potentially pathogenic variants in other genes with known association with neurodevelopmental disorders were also present.

Written informed consent was obtained from the parents of participants according to approved protocols by the Baylor College of Medicine Institutional Review Board.

Clinical data was retrospectively reviewed by a board-certified neurologist (JLH) and included personal and family history (including antenatal/perinatal history) and all history of care for seizures/epilepsy, development and behavior, physical examination, and neurologic examination. In addition, all historical neuroimaging (computed tomography, CT, or magnetic resonance imaging, MRI) was reviewed by pediatric neuroradiologists at our institution. All scalp electroencephalograms with a minimum of 21 electrode recordings in a standard 10–20 distribution were reviewed by board-certified neurophysiologists (RC and MQ). As an exploratory function, all traits within the standard clinical read were collected (background continuity, posterior dominant rhythm, symmetry and synchrony, interictal epileptiform discharges, ictal discharges, and other abnormalities not associated to epileptogenesis).

A neurodevelopmental evaluation was performed on all patients as part of the clinical evaluation. All neurodevelopmental data was reviewed by board-certified neurodevelopmental pediatricians (SV and SR). Whenever possible, clinical information was obtained with standardized testing by utilizing Gesell development schedules for gross motor (GM) assessment and The Capute Scales for infant development for visual-perceptual/fine motor (VP/FM) assessment and language/speech (LANG) assessment. The Capute Scales utilize two domains of assessment for this purpose: a Cognitive Adaptive Test that provides independent developmental quotients for VP/FM skills and can be extrapolated to evaluate for early non-verbal problem-solving skills and early adaptive skills and a Clinical Linguistic and Auditory Milestones Scale, which assesses early life receptive and expressive language. In addition, the combination of scores from The Capute Scales provides a Full-Scale Developmental Quotient (FSDQ) [18]. Whenever no standardized testing was formally obtained, clinical data was assessed to identify and determine a specific developmental quotient (DQ) at a minimum for the three domains described previously. In addition, we specifically evaluated key developmental milestones in patients harboring deleterious *SYNGAP1* variants: ages for sitting unaided, walking independently, saying first word, scribbling, and using utensils by parental report.

Descriptive statistics were obtained for all variables, and data was classified into categorical and ordinal variables whenever relevant. Given the nature of this retrospective study, a variable number of time-points were available for analysis in this cohort. Data from patients in whom multiple data collection time-points were available (corresponding to follow-up visits at least 1 year apart) was utilized as independent data (i.e., in isolation) for the purpose of inferential data analysis. Select variables were tested for potential associations (by Fisher’s exact test) and correlations (by Pearson’s correlations) to determine possible biomarkers of clinical utility in assessing severity, progression, or prognosis. Normal distribution of all data was confirmed using the Shapiro-Wilk test for normalcy.

## Results

A total of 15 subjects were identified in the study period, of which eight (53%) were male and seven (47%) were female. Mean age at diagnosis was 65.9 months (range 28–174 months).

### Genetics

**Fig. 1:**
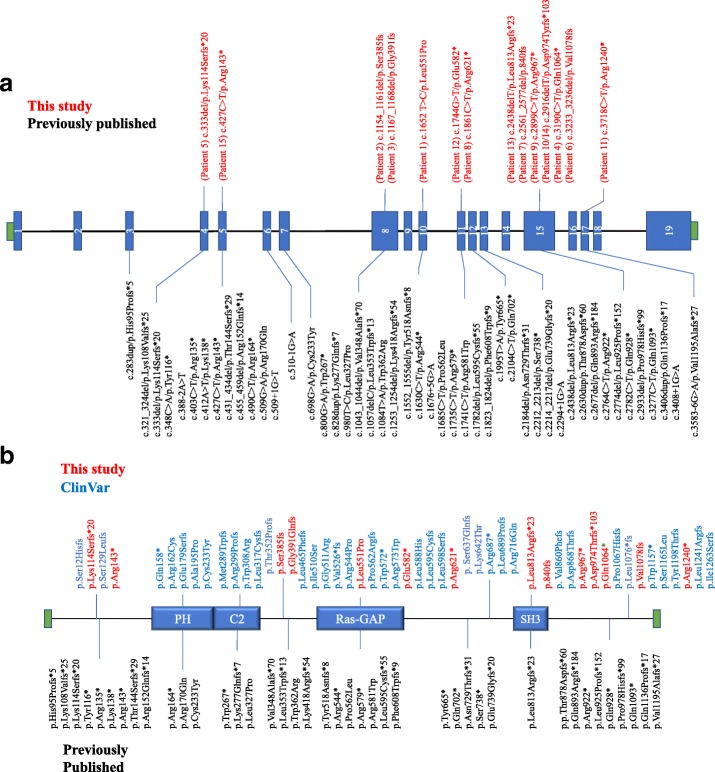
Pathogenic variants in *SYNGAP1*. **a** Diagram of *SYNGAP1* gene. Pathogenic variants reported in this study above the gene. Previously published pathogenic variants below the gene. Repeat variants between this study and previously published variants represent separate, unique individuals to the best of our knowledge. **b** Diagram of SynGAP protein. Pathogenic protein variants from this study and ClinVar above the protein. Previously published protein variants below the protein. Repeat variants between this study and previously published variants represent separate, unique individuals to the best of our knowledge

Fifteen individuals with seventeen *SYNGAP1* variants which were discovered via clinical sequencing panels or exome sequencing are presented (Fig. 1, Table 1 and Additional file 1: Table S1). We compiled our data set, previously published studies, and likely pathogenic or pathogenic variants in *SYNGAP1* found in ClinVar (Additional file 1: Table S1). From this combined dataset, there are seventeen variants which have been reported in our cohort or in previous publications that have been reported in ClinVar. Based on the date of submission in ClinVar compared to the date of publication for the corresponding paper, sixteen of these variants appear to be unique cases instead of repeated reports, with some variants being reported on multiple occasions in ClinVar. These data suggest that there might be loci within the *SYNGAP1* gene that are prone to mutation. For example, one variant (p.Pro562Leu) reported in Mignot et al. [16] has been reported four times in ClinVar and lies within the RASGAP domain of SynGAP. Additionally, another variant (p.Arg143*) reported in two publications [15, 16] was also reported four times in ClinVar.

**Table 1:** Genotypes and phenotypes of *SYNGAP1* cohort

Subject	Genetic mutation	Age at diagnosis (months)	Age at seizure onset	EEG—intermittent rhythmic delta activity	EEG—interictal epileptiform activity	Cognitive/developmental impairment*	MRI abnormalities
1	c. 1744G>T (p.E582X), c.2693C>G (p.S898C), c.2305C>G (p. L769 V)	32	23	–	Generalized spike-slow wave; evolved to multifocal epileptiform activity	Moderate	Mildly simplified frontal lobe gyral pattern
2	c.1652 T>C (p.L551P); chr6: 33406672 (NM_006772.2)	28	18	Occipital	Generalized posterior dominant spike and wave discharges	Severe	T2 white matter subinsular, periatrial, and subcortical hyperintensities
3	c.427C>T (p.Arg143*)	45	24	–	Generalized and focal polyspike and wave and spike activity	Moderate	Small developmental venous anomalies
4	c.1154_1161del (p.S385 fs)	66	12	Generalized, maximal left hemisphere	Generalized polyspike and wave and spike and wave activity	Profound	–
5	c. 1167-1168del (p.G391 fs)	174	120	Generalized posterior dominant	Generalized spike and wave discharges and multifocal spikes	Profound	–
6	c.3190C>T (p. Q1064X), 3p12 loss	36	30	Generalized posterior dominant	Generalized spike and wave discharges	Profound	–
7	c.333del (p.L114Sfs20)	81	30	–	Generalized and multifocal spike and polyspike and wave discharges	Mild	–
8	c.3233_3236del (p.V1078 fs)	80	12	–	Multifocal spikes	Profound	Nonspecific punctate white matter hyperintense foci
9	c.2561-2577del (p.840 fs)	65	17	Occipital and frontocentral	Generalized and multifocal spike and polyspike and wave discharges	Profound	Nonspecific punctate white matter hyperintense foci
10	c.1861C>T (p.R621X)	90	48	–	Generalized spike and wave discharges	Profound	Chiari 1; thick corpus callosum
11	c. 2899 C>T (p.R967X)	36	37	Posterior and left mid-temporal	Generalized posterior dominant spike and wave discharges	Profound	Chiari 1
12	c.2916delT (p.D974Tfs*103)	108	103	Right temporo-occipital	Generalized and focal right occipital spikes	Severe	–
13	c.3718C>T (p.R1240X)	55	62	Parieto-occipital bilateral	Generalized spikes and polyspikes; focal left occipital spikes	Severe	T2 hyperintensities in bilateral centrum semiovale
14	c.2438delT (p.L813Rfs*23)	67	24	–	Generalized posterior dominant spike and wave discharges	Severe	–
15	c.2916delT (p.D974Tfs*103)	60	24	Posterior bilateral	–	Moderate	–

*Based on approximate standard scores for Full-Scale Developmental Quotient: mild developmental/intellectual disability (DD/ID) 50–69; moderate DD/ID 35–49; severe DD/ID 20–34; profound DD/ID < 20

We then compared this compiled dataset of pathogenic variants to the Exome Aggregation Consortium (ExAC) database of control exomes from individuals without neurodevelopmental disorders [19]. None of these variants were represented in the ExAC database. Furthermore, *SYNGAP1* has a probability of loss of function intolerance (pLI) of 1.0 in ExAC, demonstrating a high probability that LoF variants cause a severe phenotype precluding inheritance of deleterious variants. The *Z* score of deviation from expected allelic frequency for missense mutations in *SYNGAP1* is 7.15 (539.6 expected variants, 200 observed), again demonstrating intolerance for deleterious variants. We also examined the gnomAD database to determine if any of our patient variants were in this database of 123,136 exomes and 15,496 genomes from neurotypical individuals. One variant (p.Ser898Cys) was present in both gnomAD and a patient from our cohort as well as being predicted as deleterious through algorithms CADD, PolyPhen2, and SIFT. However, this patient had two additional variants in *SYNGAP1* (p.Glu582*, p.Leu769Val), the former of which we believe to be causative.

We investigated the pathogenicity of missense variants in our data set and in previously published data sets by using predictive algorithms CADD, PolyPhen2, PROVEAN, and SIFT. In CADD, each variant is assigned an evolutionary action (EA) score which correlates with loss of protein function [20]. In the combined missense variant data set from all sources (ClinVar, previous publications, and our data), thirteen out of twenty variants had an EA score close to or above 80, strongly suggesting deleterious impact on protein function (Additional file 1: Table S1). In PolyPhen2, eighteen out of twenty missense variants were predicted to be “probably damaging” with the other two variants predicted to be “possibly damaging.” PROVEAN predicted all but four missense variants as being “deleterious,” and SIFT predicted all but two missense variants as being “damaging.” These missense variants were observed throughout SynGAP, three in the PH domain, two in the C2 domain, nine in the RASGAP domain, and the remaining six variants interspersed throughout the protein.

### Neurophysiologic manifestations

**Fig. 2:**
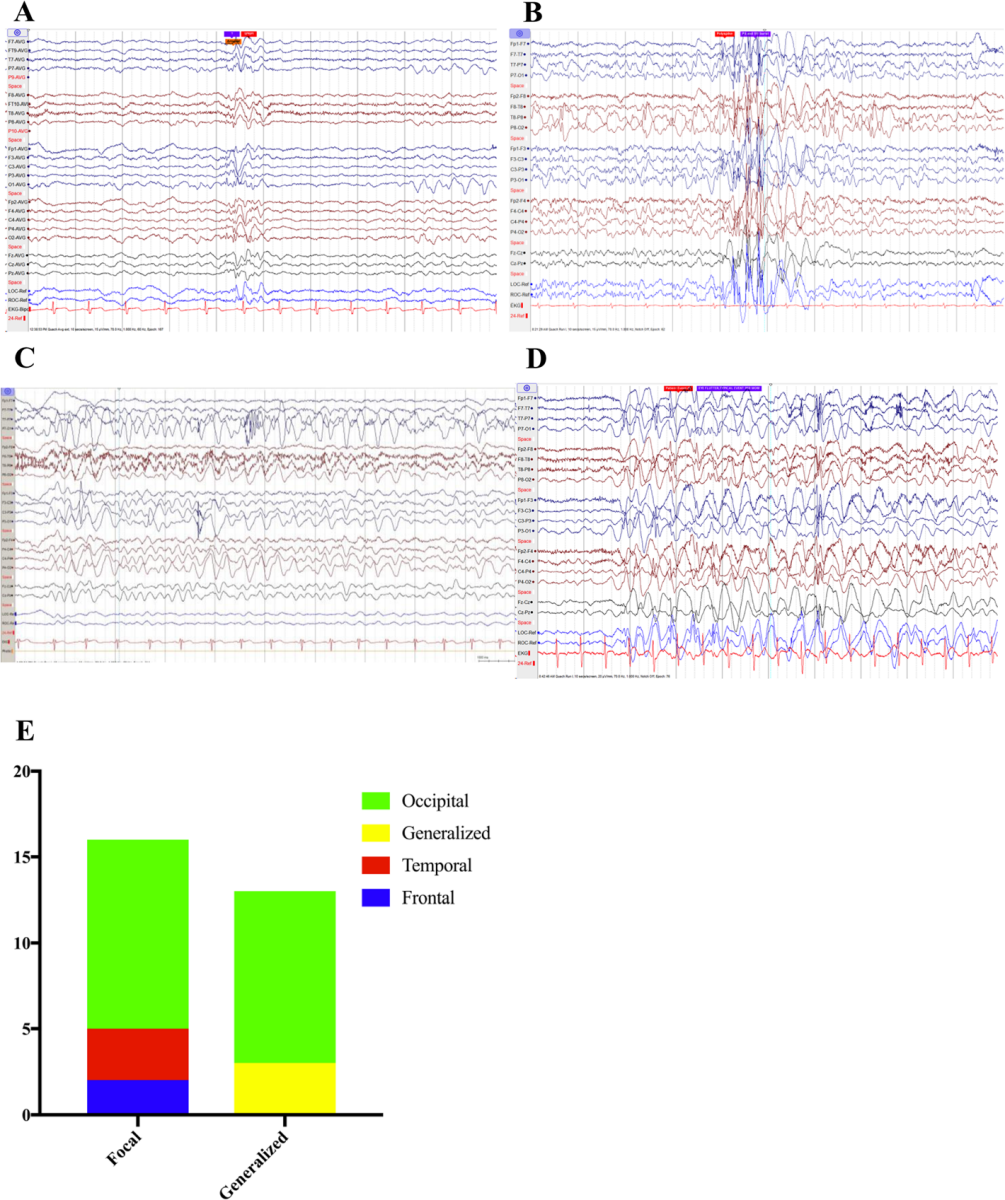
Electrographic and epileptic features in patients with *SYNGAP1* pathogenic variants. **a** Example electroencephalogram containing occipital spikes from patient with pathogenic *SYNGAP1* variant. **b** Example electroencephalogram containing polyspikes from patient with pathogenic *SYNGAP1* variant. **c** Example electroencephalogram containing rhythmic delta waves from patient with pathogenic *SYNGAP1* variant. **d** Example electroencephalogram containing seizure activity from patient with pathogenic *SYNGAP1* variant. Patient’s seizure was characterized by behavioral arrest. **e** Quantification of interictal epileptiform activity in patients with *SYNGAP1* pathogenic variants

At least one scalp EEG was performed on all patients. Epileptiform activity was captured in fourteen of fifteen individuals with generalized discharges being more common than focal (Table 1). Occipital epileptiform discharges were substantially more common than from other areas (Fig. 2a, e). The epileptiform discharges took the form of polyspikes as well as spike and slow wave (Fig. 2b). Epileptiform activity was potentiated in five children with sleep onset. Slowing or absence of the posterior dominant rhythm (relative to chronologic age) was also a common manifestation on electroencephalograms (12 of 15). Only three out of eight individuals greater than 5 years old achieved an alpha rhythm (8 Hz or greater) on EEG. In addition, nine of our fifteen patients displayed intermittent rhythmic delta activity (IRDA), with the majority of those (6 of 9) having occipital predominance (Fig. 2c).

### Epilepsy

All fifteen individuals in our cohort with germline *SYNGAP1* pathogenic variants were diagnosed with epilepsy. The mean age of onset for seizures was 38 months ± 32 (SD) with a range of 12 to 120 months. The most common seizure semiology was atypical absence (9 of 15) (example in Fig. 2d), followed by absence (4 of 15), then generalized tonic-clonic and atonic (three each), and finally two patients with focal onset seizures.

### Developmental progression

**Fig. 3:**
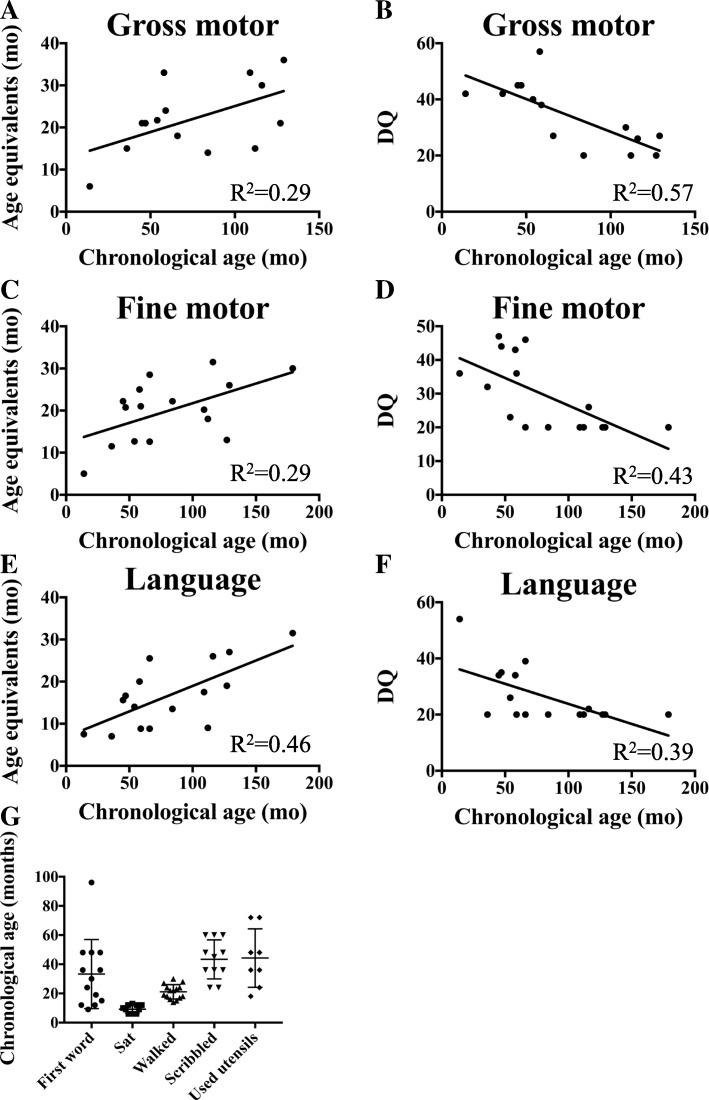
Developmental correlations with chronological age. **a** Gross motor age equivalents plotted against chronological age. **b** Gross motor developmental quotient (DQ) plotted against chronological age. **c** Fine motor age equivalents plotted against chronological age. **d** Fine motor DQ plotted against chronological age. **e** Language age equivalents plotted against chronological age. **f** Language DQ plotted against chronological age. **g** Age at which select developmental milestones occur in *SYNGAP1* patient cohort. Mean ± standard deviation

Developmental testing data was obtained for all individuals (Additional file 2: Table S2). Standardized testing for gross motor development was performed for eight of our fifteen children with deleterious germline *SYNGAP1* mutations using the Gesell development schedules [21]. Significant delay was noted in all individuals. Three individuals had repeated testing over yearly (or greater) intervals. For all of these individuals, there was plateauing of development at approximately 60 months of age. Overall there was only a suggestion of a mild correlation between chronological age and age equivalents for gross motor development of this cohort (Fig. 3a), while there was a moderate negative correlation between gross motor developmental quotient and chronological age (Fig. 3b); however, neither reached statistical significance.

Visual-perceptual/fine motor skill (VP/FM) was evaluated by the Cognitive Adaptive Test (CAT) component of The Capute Scales [18] for ten subjects with pathogenic *SYNGAP1* variants on at least one occasion. Similar to gross motor skills, VP/FM skills only demonstrated a mild positive correlation that was not statistically significant between age equivalents and chronological age (Fig. 3c), while also demonstrating a trend toward mild to moderate negative correlation between developmental quotient and chronological age (*p* = 0.1) (Fig. 3d).

Language development was evaluated for ten subjects with pathogenic *SYNGAP1* variants using the Clinical Linguistic and Auditory Milestone Scale (CLAMS) component of The Capute Scales [18]. In contrast to gross motor and VP/FM development, language skills trended toward a moderate positive correlation between age equivalence and chronological age (*p* = 0.07) (Fig. 3e). There was a mild trend toward a negative correlation between language developmental quotient and chronological age (*p* = 0.142) (Fig. 3f).

We specifically evaluated the timing of acquisition of key developmental milestones in patients harboring deleterious *SYNGAP1* variants: ages for sitting unaided, walking independently, saying first word, scribbling spontaneously, and using utensils. All of these were found to be significantly delayed in our cohort of children (Fig. 3g).

**Fig. 4:**
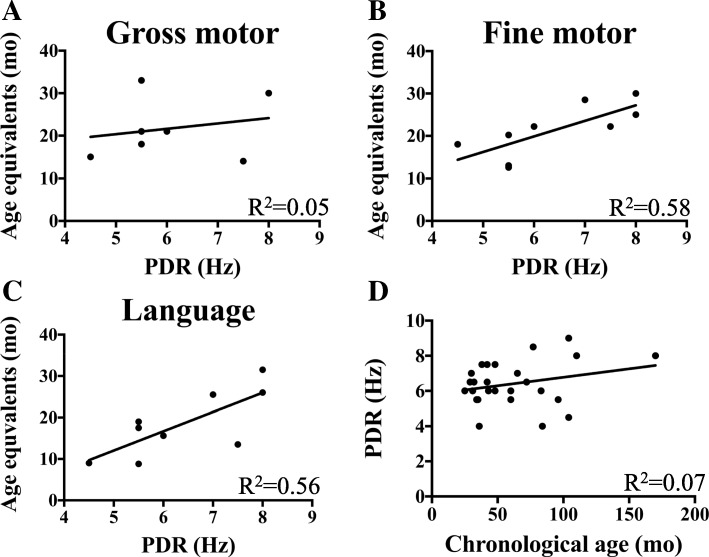
Developmental correlations with posterior dominant rhythm (PDR). **a** Gross motor age equivalents plotted against posterior dominant rhythm (PDR). **b** Fine motor age equivalents plotted against PDR. **c** Language age equivalents plotted against PDR. **d** PDR plotted against chronological age

When evaluating neurophysiologic data and development, only VP/FM (*p* = 0.1) and language skills (*p* = 0.114) displayed a trend toward moderate correlation between age equivalents and posterior dominant frequency (Fig. 4a–c). The frequency of the posterior dominant rhythm (PDR) itself did not correlate with age in its expected chronologic-developmental maturation; this suggests that the uncovered development-PDR correlations were not simply due to changes in PDR with age (Fig. 4d). There was no association between the presence of IRDA and severity of developmental disability in any domain (mild/moderate vs. severe/profound disability, *p* = 0.235).

### Other neurologic manifestations

Strabismus was present in over 60% of our patients with over 60% of those patients requiring corrective surgery. Sleep abnormalities were reported in two thirds of our patients with insomnia manifested as nighttime awakenings being the predominant issue. Only one patient was identified as having obstructive sleep apnea. This suggests that for most patients, the insomnia was primary. Neurologic examination revealed low muscle tone in one third of our patients and elevated tone in only one. While general motor strength was normal in these patients, significant ataxia was identified in 21%.

### Neuroimaging characteristics

Magnetic resonance imaging (MRI) of the brain was obtained in all patients in our cohort at an average age of 44.6 months ± 30 months (SD). Seven patients (47%) had normal neuroimaging. Among those with an abnormal MRI, nonspecific white matter hyperintensities were observed on T2/FLAIR sequences in four patients with variable location including frontal lobes, centrum semiovale, subinsular, and periatrial. One patient had an isolated Chiari I malformation, whereas another had Chiari I and a thickened corpus callosum. A predominantly frontal, mildly simplified gyral pattern was present in one patient, and another had several small developmental venous anomalies (Additional file 3: Figure S1).

### Neurobehavioral manifestations

Behavioral abnormalities were amply described in our cohort. Aggressive behavior toward caregivers and siblings was reported in 60% of our patients at any point in time. Self-injurious behavior of any nature was reported in a third of our cohort, described most frequently as biting oneself. Disruptive hyperactivity was also reported in a third of our patients. Eleven of fifteen (73%) have been diagnosed with autism spectrum disorder by a medical provider.

## Discussion

Deleterious variants in *SYNGAP1* have been identified in a variety of phenotypically defined cohorts including syndromic/non-syndromic intellectual disability, autism spectrum disorder, and epileptic encephalopathy [4, 6, 13, 15, 16]. In our cohort of children with pathogenic *SYNGAP1* variants, we corroborated developmental delay/intellectual disability and epilepsy as universal features.

Neurodevelopmental traits-global delay/disability must be analyzed considering the evolving nature of developmental test scoring. The universality and severity of neurocognitive impairment is presented in Table 1 and is derived from the assessment of developmental quotients (DQ = age equivalence/chronologic age), with severity of impairment most significant in language skills and least in gross motor skills. Our data is similar to prior cohorts in the preponderance of severe disability, especially evident at a later age [15, 16]. Our cohort displayed weak to moderate positive correlations between the developmental and chronologic age (Fig. 3a, c, e), contrasted with a weak to moderate negative correlation between developmental quotients and chronologic age (Fig. 3b, d, f). We believe this to be expected given the growing dissociation between chronologic and developmental age, without of a true plateau or regression, in development. It may be most useful in future clinical studies to utilize age equivalents in measuring developmental progression in these individuals.

Diagnosis of autism spectrum disorder was present in just under three quarters of our cohort, comparable with prior reports ranging from 50 to 80% [15, 16]. It has been proposed that while mutations in the *SYNGAP1* gene likely play a role in the pathogenesis of ASD, their presence is potentially not sufficient for its development [16]. In addition, the variable manifestations of ASD can evolve with age, suggesting that an absence of this diagnosis at a single point in time may not hold true later, and ASD may increase in prevalence with age in this population.

Prior studies have linked neurophysiologic abnormalities to developmental outcomes such as in epileptic encephalopathies and autism spectrum disorders (ASDs) [16]. Capal et al. recently suggested abnormal EEG patterns, even in the absence of epilepsy, were linked with worse developmental outcomes in children with ASD [22]. Similarly, abnormal EEG patterns have been linked to a number of other genetically defined intellectual and developmental disorders [23–25]. Based on our data, we hypothesize this might be true for patients with *SYNGAP1* mutations. First, most children in our study were found to have interictal epileptiform discharges. For the majority of our subjects, there was a posterior prominence of these discharges. These discharges were enhanced with sleep in a subset of our patients. Second, the majority of our subjects also displayed intermittent rhythmic delta activity. Third, we identified abnormally slow posterior dominant rhythm for age in the majority of our patients. The frequency of the PDR is known to increase with development typically achieving the alpha range (8–12 Hz) by 4–5 years of age. Only three of fifteen individuals in our cohort achieved an alpha frequency of their PDR despite most having an EEG after their fourth birthday (11 of 15).

One of the greatest challenges for developing targeted therapies for neurodevelopmental disorders is in identifying quantitative biomarkers that directly correspond to clinical outcomes. Indeed, lack of such biomarkers for most neurodevelopmental disorders has potentially led to disappointing results for late-stage clinical trials such as for fragile X syndrome [26]. As described above, we have identified several neurophysiologic features that warrant further investigation as potential biomarkers for disease progression identified in our cohort. The most salient findings include a moderate correlation between developmental age equivalence in language and VP/FM development with the frequency of the posterior dominant rhythm. This correlation is not simply due to maturation of the posterior dominant rhythm with age as most of our data comes after 4 years of age when the PDR has achieved maturity. Furthermore, plotting PDR frequency versus chronological age in our cohort confirmed that there was no correlation. Despite the listed descriptions of EEG anomalies in other neurodevelopmental conditions, there has not been—to our knowledge—a prior report of a correlation with PDR frequency and developmental progression for any genetically defined disorder.

Some trends began to emerge in the genetic architecture of pathogenic variants in *SYNGAP1*. First, combining our data with all previously published mutations and pathogenic variants from ClinVar, the majority of mutations fall within exons 3–17, sparing the first two (except for a single exon 1 mutation) and last two exons. Why these exons are spared is unclear but might be due in part to the extensive alternative slicing that occurs in the five prime and three prime regions of the *SYNGAP1* transcript. Second, while we have identified a small number of recurrent mutations in *SYNGAP1*, the vast majority are novel non-sense or frameshift variants confirming that patients’ variants must largely be loss of function.

Our study has multiple limitations. Since this was a retrospective study, we have performed an exhaustive data analysis to the extent permitted by clinical documentation within a specialty clinic. This has limited the number of potential time-points in a single individual that can be analyzed for all desired parameters, to establish comprehensive neurodevelopmental and neurobehavioral trajectories. Given the overall neurologic stability, patients are seldom followed more frequently than every 6 months, and we deliberately collected only time-points at least 1 year apart to allow for developmental changes to clearly emerge. Inference and conclusions regarding neurodevelopmental trajectories (progression, regression, or plateauing) are limited. Thus, these data are exploratory and hypothesis generating. Future collection in a prospective observational manner may better help standardize and enrich these findings. Given the rarity of genetically confirmed *SYNGAP1* patients, the size of our current cohort also limits potential inferences, including describing a clear genotype-phenotype correlation with any of the developmental parameters evaluated in these patients.

## Conclusions

Evaluation of neurodevelopmental progression in individuals with pathogenic *SYNGAP1* variants revealed a broad spectrum, most often ranging from moderate to severe impairment. Furthermore, the frequency of posterior dominant rhythm of EEG in these individuals revealed a trend toward correlation with developmental progression, providing a possible prognostic biomarker. Further assessment using more extensive neuropsychological cognitive/developmental evaluation in a prospective manner with larger cohorts will better define developmental progression and provide benchmarks for future targeted clinical trials for individuals with pathogenic *SYNGAP1* variants. In this regard—and given the rarity of this genetically defined neurodevelopmental disorder—expanding the number of participants in these cohorts may require inter-institutional collaborations to achieve larger sample sizes and potentially statistical significance.

## Additional files

Additional file 1: Table S1. Compiled list of all pathogenic variants. Table contains all pathogenic variants in the current report, previously published, and from the ClinVar database. (XLSX 18 kb)

Additional file 2: Table S2. Developmental and behavioral phenotypes of a *SYNGAP1* cohort. Table contains developmental and behavioral findings for each individual including clinically relevant milestones in each of gross motor, visual-perceptual/fine motor, and language domains, as well as neuropsychiatric findings including autism spectrum disorder. (DOCX 15 kb)

Additional file 3: Figure S1. MRI imaging findings. (A) Subject #1 at 34 months. Sagittal T1 (left) and coronal T2 (right) images show diffuse mildly simplified gyri, predominantly in the frontal lobes. (B) Subject #13 at 6 years and 6 months. Axial T2/FLAIR images show diffuse hyperintense signal in the bilateral centrum semiovale (left, arrows) and punctate foci of subcortical white matter hyperintensity (right, arrowheads). (C) Subject #10 at 11 years and 4 months. Sagittal T2/FLAIR images demonstrate diffusely thickened corpus callosum (arrows) and a mild Chiari I malformation (arrowheads). (PPTX 242 kb)

## Data Availability

The data sets used and/or analyzed in the current study are available from the corresponding author on reasonable request.

## Abbreviations

AMPAR: α-Amino-3-hydroxy-5-methyl-4-isoxazolepropionic acid receptor
CaMKII: Calmodulin-dependent protein kinase II
CAT: Cognitive Adaptive Test (part of The Capute Scales)
CLAMS: Clinical Linguistic and Auditory Milestone Scale (part of The Capute Scales)
EEG: Electroencephalogram
IRDA: Intermittent Rhythmical Delta Activity
mEPSC: Miniature excitatory postsynaptic currents
MRI: Magnetic resonance imaging
NMDAr: *N*-Methyl-d-aspartate receptor
PDR: Posterior dominant rhythm
VNS: Vagal nerve stimulator
