# Supplementary material for: Phenotypic characterization of individuals with SYNGAP1 pathogenic variants reveals a potential correlation between posterior dominant rhythm and developmental progression
Source: J Neurodev Disord. 2019 Aug 8;11:18. doi: 10.1186/s11689-019-9276-y (PMC6688356; doi:10.1186/s11689-019-9276-y)
Supplement: Supplementary file 2 — Table S2. Developmental and behavioral phenotypes of a SYNGAP1 cohort. Table contains developmental and behavioral findings for each individual including clinically relevant milestones in each of gross motor, visual-perceptual/fine motor, and language domains, as well as neuropsychiatric findings including autism spectrum disorder. (DOCX 15 kb) [file 11689_2019_9276_MOESM2_ESM.docx]

TABLE S2

| **Subject** | **Age at diagnosis (months)** | **Age at assessment (months)** | **Major gross motor ability (developmental age equivalent)** | **Reported Visual-Perceptual/Fine motor/adaptive ability (developmental age-equivalent)** | **Reported language/speech ability (developmental age-equivalent)** | **Full scale Cognitive/developmental impairment*** | **Neurobehavioral findings** |
| --- | --- | --- | --- | --- | --- | --- | --- |
| 1 | 32 | 50 | Walks (16 months) | Feeds with utensils (21 months) | 20 words (21 months) | Moderate | Autism spectrum disorder  Hyperactive/disruptive  Self-Injurious  Aggressive |
| 2 | 28 | 34 | Walks (18 month) | Feeds with utensils (18 month) | Nonverbal, babbles (6 months) | Severe | Autism spectrum disorder  Self-injurious  Aggressive |
| 3 | 45 | 50 | Walks; can climb stairs (23mo) | Finger feeds (20 month) | 50 words; follows two-step commands (24 months) | Moderate | Self-injurious  Aggressive |
| 4 | 66 | 120 | Walks (21 months) | Feeds with utensils; helps dress (27 months) | 4 words and 6 signs (21 months) | Profound | Autism spectrum disorder  Hyperactive/disruptive  Aggressive |
| 5 | 174 | 178 | Walks; stairs alternating feet; jumps (36 months) | Feeds with utensils (30 months) | 250 words (31 months) | Profound | Autism spectrum disorder  Hyperactive/disruptive |
| 6 | 36 | 61 | Walks (15 months) | Feeds with utensils (21 months) | Nonverbal, babbles (10 months) | Profound | Autism spectrum disorder |
| 7 | 81 | 109 | Walks (21 months) | Feeds with utensils; writes 3-letter words; recognizes letters (6 years) | 4-5 word sentences; can relay experiences; reads (6 year old) | Mild | Hyperactive/disruptive |
| 8 | 80 | 12 | Walks; stairs alternating feet (33 months) | Pincer grasp; finger feeds (12 month) | 10-15 words; follows one step commands (21 month) | Profound | Autism spectrum disorder  Aggressive |
| 9 | 65 | 112 | Walks (21 months) | Finger feeds (22 month) | Nonverbal; 5 signs (12 month) | Profound | Autism spectrum disorder  Aggressive |
| 10 | 90 | 142 | Walks unaided (24 months) | Feeds with utensils (26 months) | 10-15 words; 2 word sentences (27 months) | Profound | Autism spectrum disorder |
| 11 | 36 | 84 | Walks (14 months) | Finger feeds (22 months) | 1-2 Words (14 months) | Profound | Autism spectrum disorder  Aggressive |
| 12 | 108 | 130 | Walks (21 months) | Feeds with utensils (36 months) | 100 words, 3-word sentences (30 months) | Severe | Autism spectrum disorder  Hyperactive/disruptive  Self-injurious  Aggressive |
| 13 | 55 | 116 | Walks; jumps (33 months) | Feeds with utensils (31 months) | 250 word vocabulary (26 months) | Severe | Autism spectrum disorder  Hyperactive/disruptive |
| 14 | 67 | 84 | Walks (18 months) | Finger feeds (18 months) | 20-30 words (24 months) | Severe | Autism spectrum disorder  Hyperactive/disruptive  Self-injurious  Aggressive |
| 15 | 60 | 65 | Walks; can climb stairs (30 months) | Feeds with utensils (28 months) | 50 words; 2-step commands (29 months) | Moderate | - |

* Based on approximate standard scores for Full Scale Developmental Quotient (VP/FM + Language): Mild Developmental/intellectual disability (DD/ID) 50-69; Moderate DD/ID 35-49; Severe DD/ID 20-34; Profound DD/ID <20
