# Supplementary figures and images for: Phenotypic characterization of individuals with SYNGAP1 pathogenic variants reveals a potential correlation between posterior dominant rhythm and developmental progression
Source: J Neurodev Disord. 2019 Aug 8;11:18. doi: 10.1186/s11689-019-9276-y (PMC6688356; doi:10.1186/s11689-019-9276-y)

## Slide 1
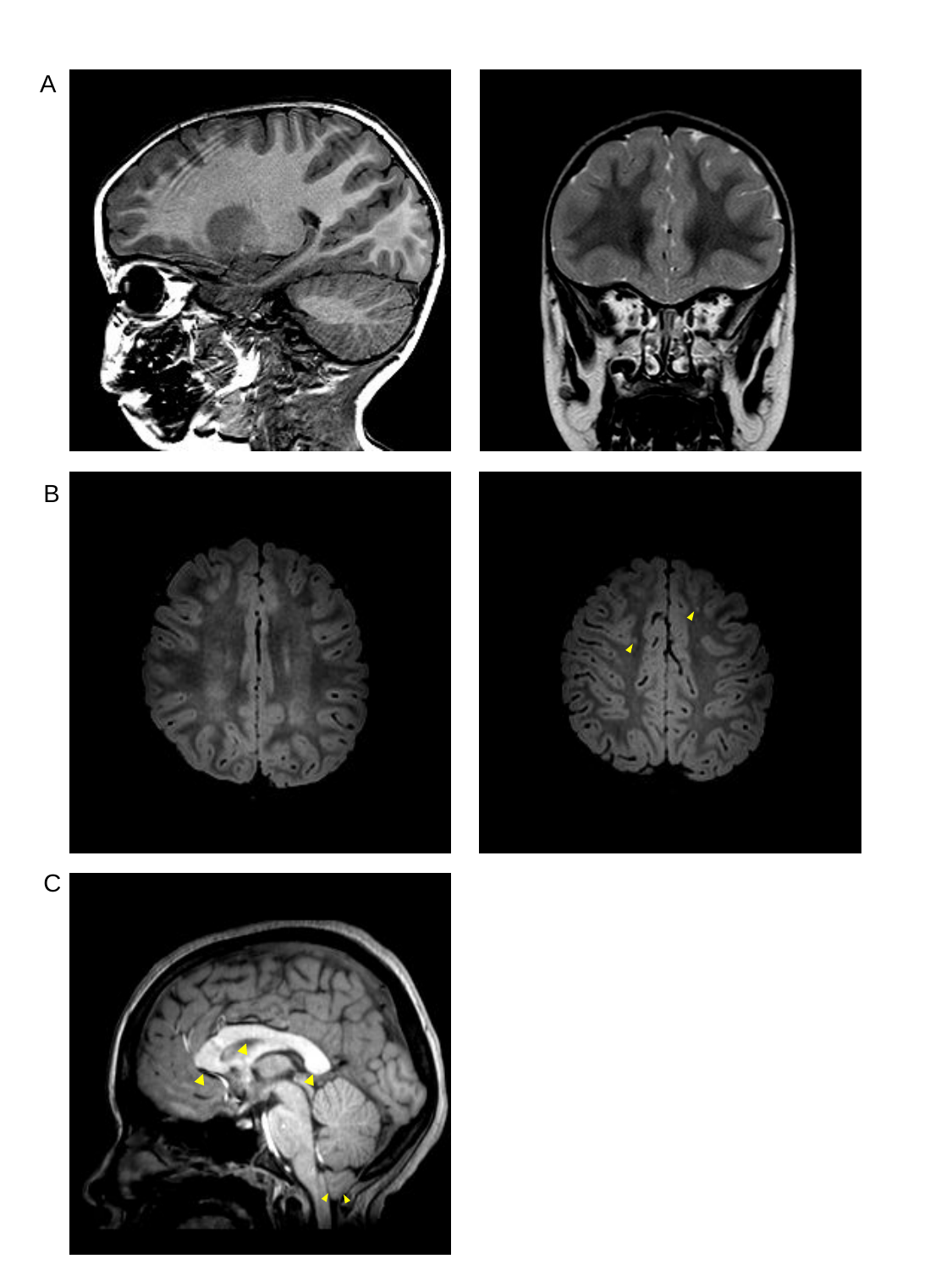

A
B
C

Supplement: Supplementary file 3 — Figure S1. MRI imaging findings. (A) Subject #1 at 34 months. Sagittal T1 (left) and coronal T2 (right) images show diffuse mildly simplified gyri, predominantly in the frontal lobes. (B) Subject #13 at 6 years and 6 months. Axial T2/FLAIR images show diffuse hyperintense signal in the bilateral centrum semiovale (left, arrows) and punctate foci of subcortical white matter hyperintensity (right, arrowheads). (C) Subject #10 at 11 years and 4 months. Sagittal T2/FLAIR images demonstrate diffusely thickened corpus callosum (arrows) and a mild Chiari I malformation (arrowheads). (PPTX 242 kb) [file 11689_2019_9276_MOESM3_ESM.pptx]
